# Comparison of failure modes and effects analyses and time for brachytherapy ring and tandem applicator digitization between manual and solid applicator source placement methods

**DOI:** 10.1002/acm2.14336

**Published:** 2024-04-25

**Authors:** Adia L. Holtman, Dominic J. DiCostanzo, Charles A. Zimmerman, Gavin Graeper, Jeffrey Woollard, Daniel F. Christ, Ashley J. Cetnar

**Affiliations:** ^1^ Department of Radiation Oncology The Ohio State University Columbus Ohio USA

**Keywords:** applicator digitization, brachytherapy, ring and tandem, solid applicator

## Abstract

**Purpose:**

Ring and tandem (R&T) applicator digitization is currently performed at our institution by manually defining the extent of the applicators. Digitization can also be achieved using solid applicators: predefined, 3D models with geometric constraints. This study compares R&T digitization using manual and solid applicator methods through Failure Modes and Effects Analyses (FMEAs) and comparative time studies. We aim to assess the suitability of solid applicator method implementation for R&T cases

**Methods:**

Six qualified medical physicists (QMPs) and two medical physics residents scored potential modes of failure of manual digitization in an FMEA as recommended by TG‐100. Occurrence, severity, and detectability (OSD) values were averaged across respondents and then multiplied to form combined Risk Priority Numbers (RPNs) for analysis. Participants were trained to perform treatment planning using a developed solid applicator protocol and asked to score a second FMEA on the distinct process steps from the manual method. For both methods, participant digitization was timed. FMEA and time data were analyzed across methods and participant samples

**Results:**

QMPs rated the RPNs of the current, manual method of digitization statistically lower than residents did. When comparing the unique FMEA steps between the two digitization methods, QMP respondents found no significant difference in RPN means. Residents, however, rated the solid applicator method as higher risk. Further, after the solid applicator method was performed twice by participants, the time to digitize plans was not significantly different from manual digitization

**Conclusions:**

This study indicates the non‐inferiority of the solid applicator method to manual digitization in terms of risk, according to QMPs, and time, across all participants. Differences were found in FMEA evaluation and solid applicator technique adoption based on years of brachytherapy experience. Further practice with the solid applicator protocol is recommended because familiarity is expected to lower FMEA occurrence ratings and further reduce digitization times.

## 1 INTRODUCTION

**FIGURE 1:**
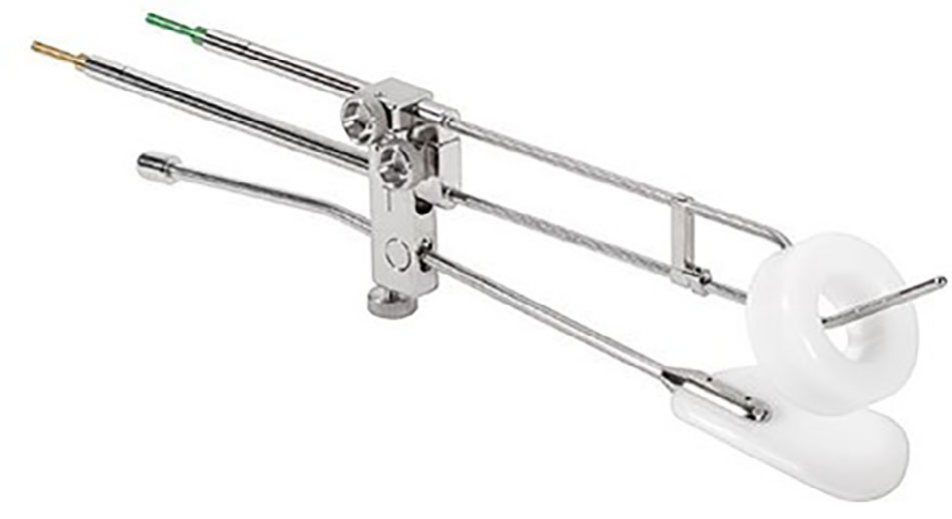
CT/MR ring and tandem applicator sets (Mick^®^ HDR applicators).^1^

Brachytherapy is a type of radiation oncology procedure which employs radioactive sources placed at short distances to treat disease. Intracavitary or interstitial brachytherapy is often useful for the treatment of gynecological tumors. For cervical cancer, the two‐part ring and tandem (R&T) applicator shown in Figure 1 is commonly used to guide a high dose rate (HDR) source.^1^ The rod‐shaped tandem is positioned centrally in the uterus, and the circular ring is positioned in the vagina abutting the cervical fornices.^2^ After applicator insertion by the physician, imaging is performed to visualize the orientation of the applicator with respect to the disease. Digitization of the applicator on the acquired scan is necessary to accurately display the radiation dose distribution to targets and organs at risk (OARs) prior to brachytherapy treatment.

There lacks a universally adopted method for digitizing the R&T. Published methods include manual (or direct) and solid applicator digitization.^3^ Due to the unclear superiority of a particular method, AAPM/GEC‐ESTRO Task Group‐236 is currently involved in developing a workflow and consensus on intracavitary brachytherapy applicator digitization among other aims.^3^, ^4^, ^5^ Further, some brachytherapy teams are exploring novel approaches beyond the well‐documented digitization methods to offer a superior option.^6^, ^7^, ^8^ As these approaches are not available presently, this study focuses on the manual and solid applicator digitization methods.

At our institution, the current protocol for R&T applicator digitization is the manual method. This process includes aligning the CT viewing planes to the R&T followed by individually defining applicator structures in the treatment planning software to establish the tip and extent of each applicator. Similarly, solid applicators can be used to achieve digitization. This solid applicator method includes inserting a 3D computer model of the applicator from an applicator library followed by rotation and translation of the applicator to the CT image. This method is currently utilized at our center for vaginal cylinders, an applicator with simpler geometry. One advantage of the solid applicator method arises from the pre‐defined placement of dwell positions. This geometric constraint prevents inaccurate deviations in dwell positions from the shape of the tandem or curve of the ring reducing variation in dose distribution.^3^

It is important to consider potential failure modes in brachytherapy treatment planning such as dose estimate inconsistencies because detectable brachytherapy failure modes have led to patient harm in the past.^9^ Regarding applicator digitization, rotational or translational displacements from the true position showed up to 6% changes in dose distribution to essential OARs per millimeter of deviation.^10^ Further, brachytherapy treatment occurs in real‐time with multiple team members including physicians, qualified medical physicists (QMP),^11^ and other clinical personnel and may include additional concerns if the patient's treatment utilizes anesthesia. Thus, ensuring that treatment planning is accurate and timely is critical for high‐quality care. If the time of digitization is the same or faster with the new method (i.e., non‐inferior), this would be an important consideration for patient safety and the time commitment of health professionals.

Similar to previous research on intracavitary, gynecological brachytherapy risks, Failure Modes and Effects Analyses (FMEAs) were utilized in this study for quantification of failure effects.^12^, ^13^ FMEAs are a risk assessment strategy recommended by the AAPM Task Group 100 (TG‐100) to understand workflow variations and potential consequences.^14^, ^15^ It is recommended that regular risk analyses are performed to bring awareness to failure modes and guide protocol improvements.^9^ FMEA considers the workflow steps in terms of occurrence, severity, and detectability (OSD) of errors.^14^ For this reason, it is considered best practice to base FMEAs on process maps of clinical protocol.^15^

This report includes process maps and FMEAs for both the manual method used at our institution for R&T applicator digitization as well as for an alternative solid applicator protocol. To our knowledge, this consists of the first reported assessment of brachytherapy protocols using a comparison of FMEAs. Additional aims of this study include quantifying time differences between the digitization methods and exploring variations in FMEA ratings and digitization times based on brachytherapy experience.

## 2 METHODS

### 2.1 Overview of methods

**FIGURE 2:**
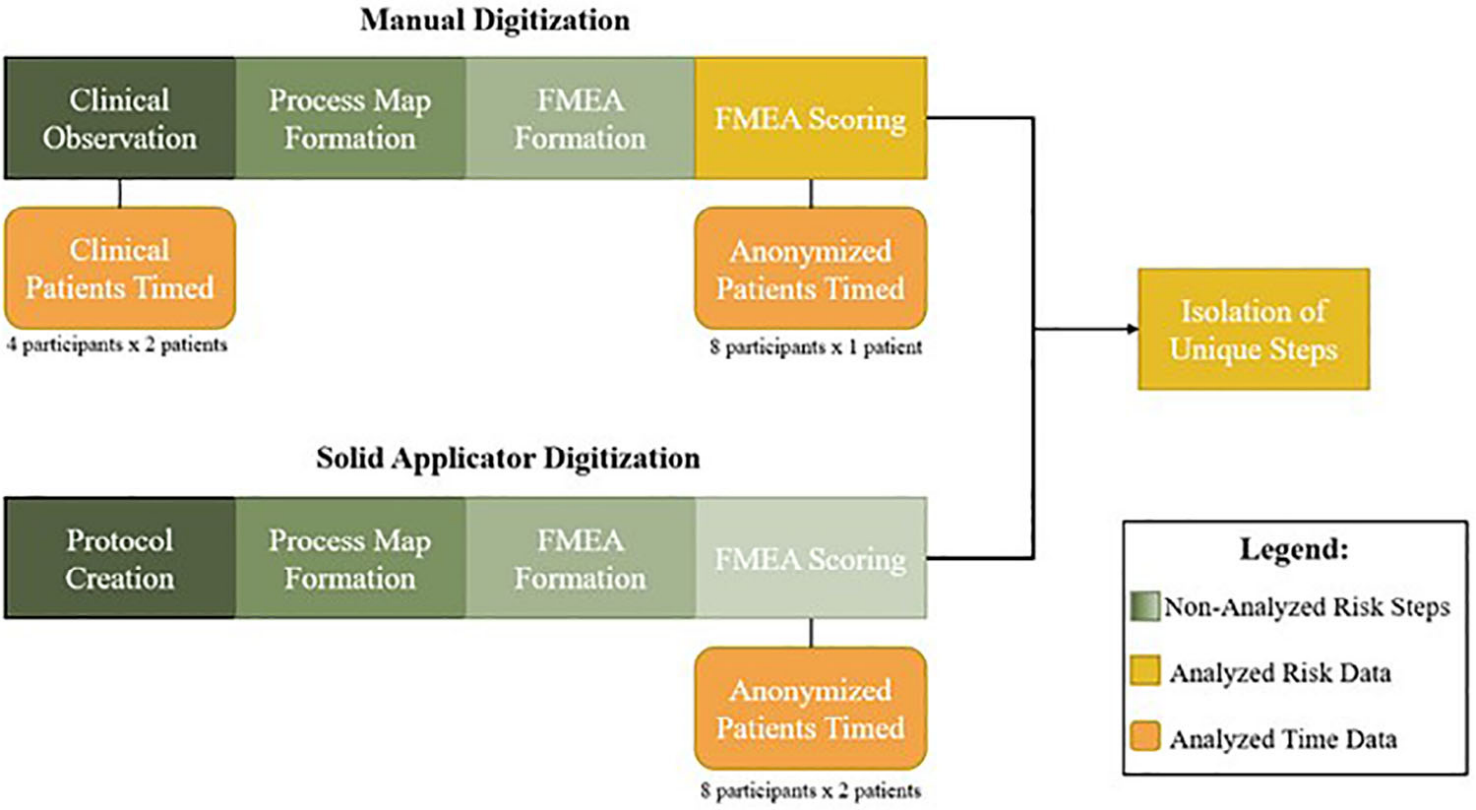
Visualized study data collection steps across both digitization methods. This includes risk (green and yellow rectangles) and time (orange, rounded rectangles) data.

This comparative R&T brachytherapy study used the Varian Eclipse Treatment Planning System for Brachytherapy Planning (version 16.01.10), BrachyVision™. Digitized applicators were Mick Radio‐Nuclear Instruments, CT/MRI Ring, and Tandem Titanium Applicator Sets.^1^ Risk data was collected through FMEAs for both methods and time of digitization was recorded for additional analysis. The course of data collection for this study is illustrated in Figure 2.

For the manual method, clinical shadowing and timing of participants were performed concurrently. This observation informed the creation of a process map and FMEA. For solid applicator digitization, the process began with generating a protocol, followed by the same steps of process map and FMEA creation. For each method, FMEAs were scored by the same participants and timing on anonymized patients was performed. The manual digitization FMEA was analyzed independently, followed by the analysis of the unique steps between the two methods.

### 2.2 Participant and patient samples

The participants of this study included all medical physicists at the institution who had both recent brachytherapy experience (i.e., within the previous year) and who would be available for the extent of the study (i.e., both FMEAs). Of the eight total participants, six were QMPs and two were medical physics residents. Additional information on participant demographics was collected during the study.

Four participants were observed in‐clinic for R&T digitization for four unique patients (eight total fractions), but no identifiable personal health information was recorded about these patients for this study. These observations solely provided information on the R&T digitization process and times in a clinical setting. Additionally, eight anonymized patients were randomly selected for use in this study from a cohort of de‐identified R&T patients over the age of 18 who were treated between 1/1/2010 and 7/3/2021 at the institution. This data use was approved by the Institutional Review Board (IRB) at our institution (2021C0132).

### 2.3 Solid applicator protocol creation

A digitization protocol was developed for the solid applicator method used in this study as an alternative to manual digitization. The aim of the solid applicator protocol was to maintain similar verification steps to the current process while offering reduced inter‐user variability. The protocol primarily utilizes Rotate Display, New Solid Applicator, Move/Rotate Applicator, and Line Profile tools in BrachyVision to align the 3D model to CT images. Step‐by‐step protocol with pictures can be found in the Supplementary Materials.

### 2.4 Process map and FMEA formation

Across clinical observations, the current workflow of R&T applicator digitization was compiled into a comprehensive process map according to TG‐100 implementation recommendations.^15^ This process map includes both applicator digitization and verification by the same participant. An FMEA was subsequently created by expanding on the process map steps to potential failure modes and causes. During process map and FMEA development, study participants were consulted for feedback in finalizing steps as well as failure modes and effects. A second process map and associated FMEA were similarly created for the solid applicator method. Process maps and FMEAs with representative data can be found in the Supplementary Materials.

### 2.5 FMEA scoring

#### 2.5.1 Manual digitization FMEA

QMPs and medical physics residents were asked to score the OSD parameters of each failure mode with a templated FMEA Excel sheet for the study. Participants were provided with a short introduction on FMEA methodology by the lead investigator as well as the following reference documents: the associated process map, a table explaining the end effects, and scoring charts based on TG‐100 recommendations.^14^ The reference sheets provided as well as an example manual FMEA sheet with average ratings can be found in the Supplementary Materials. Participants were permitted to ask any clarifying questions throughout the process of scoring, which the lead investigator answered verbally and/or visually, through applicator digitization on an anonymized patient from the cohort.

#### 2.5.2 Training and solid applicator digitization FMEA

Before the second FMEA, participants were introduced to solid applicator protocol. Participants were trained on past R&T patients from the anonymized cohort. For each study participant, the lead investigator demonstrated the solid applicator protocol on that participant's unique Practice Treatment Plan assignment in Table 1. The protocol and solid applicator process map were provided for participants during the training. Since physicists have varying experience with the solid applicator workflow, participants were given the opportunity to ask questions, observe failure modes, and experiment with the method themself on their Practice Treatment Plan. Feedback was provided by the lead investigator as requested. The participants were then asked to perform solid applicator digitization according to the protocol on two new patients based on the participant's unique Treatment Plan 1 and Treatment Plan 2 assignments in Table 1.

**TABLE 1:** De‐identified participants (1–8) and associated anonymous patient (A–H) treatment plans for digitization.

Participant identification number	Practice treatment plan	Treatment Plan 1	Treatment Plan 2
1	A	B	C
2	B	C	D
3	C	D	E
4	D	E	F
5	E	F	G
6	F	G	H
7	G	H	A
8	H	A	B

After these digitization tasks, participants were asked to provide scores to the solid applicator FMEA with the same reference documents as the first FMEA. The reference sheets as well as an example solid applicator FMEA sheet with average ratings can be found in the Supplementary Materials. To avoid comparison or bias, the two FMEAs were completed approximately a week apart for all participants. Further, since the methods contain overlapping steps, participants were only asked to score the unique steps of the solid applicator method. Physicists were offered the opportunity to re‐rank any duplicate steps they felt would be different between the methods, but no participants chose to do so.

### 2.6 Time to digitize applicators

#### 2.6.1 In‐clinic manual timing

Manual R&T applicator digitization was timed in‐clinic for three QMPs and one medical physics resident. Personnel was chosen based on participants who were on the brachytherapy rotation at the time of the study and chosen in order to maintain the same QMP‐to‐resident ratio as the entire study population (i.e., 3:1 = 6:2). Participants provided consent to be part of the timed study. Further, observations were performed over multiple patients to represent clinical case variety. The timing began at the rotation of the first viewing plane in BrachyVision to signify the beginning of digitization. (CT import and plan creation were not included at the time.) Participants were timed until the R&T applicators were both placed and adjusted accordingly with source positions assigned. To avoid additional distractions that could shift participants’ attention away from the safety of the real‐time patient case, a computer clock with minute resolution was used by the lead investigator to measure time. The convention of rounding down to the nearest minute for both start and end timepoints was adopted, incurring a variation of ±1 min. Other distractions in the clinic were unavoidable such as clinical communication with the brachytherapy team or other members of the department. Distractions were estimated to the nearest minute, and subtracted from the total time, incurring an additional variation of about ±30 s. This approximation is distinguished against stopwatch times in the analyzed data (i.e., Figure 6) by dashed, rather than solid, lines.

#### 2.6.2 Anonymous patient manual timing

Additionally, participants were asked to manually digitize using their unique Treatment Plan 1 patient from Table 1. This serves as a standard for comparison with the potential to quantify case and environment effects on digitization times. As distraction was not a pressing concern in the controlled, non‐clinical setting, these digitization times were collected using a stopwatch.

#### 2.6.3 Solid applicator timing

Participants were also timed with a stopwatch during the treatment planning using solid applicators as described in Section E.2 on their unique Treatment Plan 1 & 2 cases (Table 1). The same start and end points as manual digitization were used. Due to the novelty of the solid applicator method, participants were permitted to request feedback from the lead investigator and refer to the protocol sheet as desired during the timing study. To assess inter‐patient variability on digitization, training patients were assigned such that each case was performed by two different participants (Table 1).

### 2.7 Data analysis

#### 2.7.1 Participant experience data analysis

Demographic information including mean and standard deviation of brachytherapy experience were calculated across participants. The proportion of participants with experience performing solid applicator digitization (beyond a vaginal cylinder) was found. Further, the quantity of participants with previous exposure to the FMEA technique was determined.

#### 2.7.2 FMEA data analysis

**FIGURE 3:**
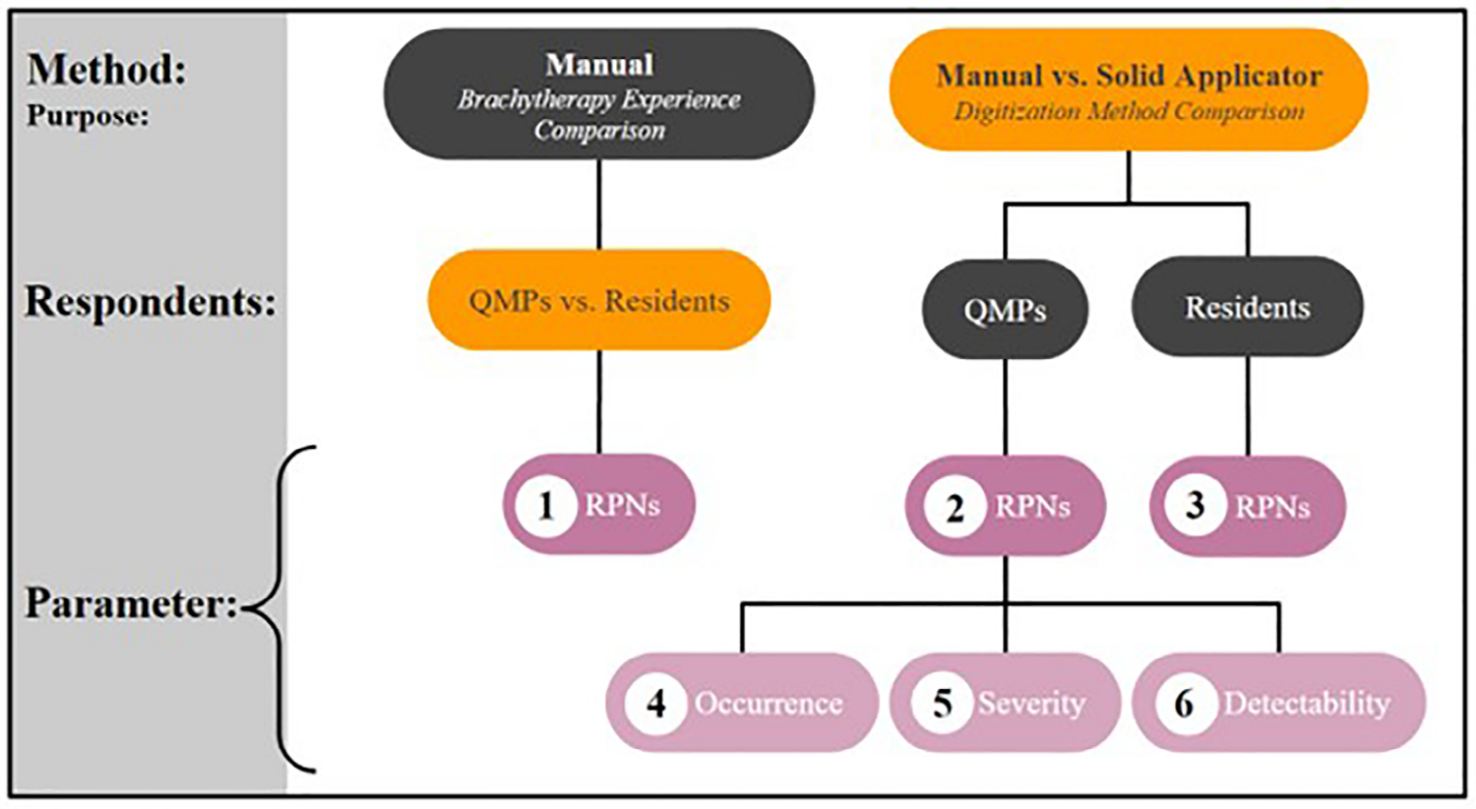
FMEA analysis tree with method, purpose, respondents, and parameter for each *t*‐test. Methods included manual and solid applicator digitization. Respondents were QMPs and residents. Parameters included RPNs and individual OSD values. *T*‐tests were performed across participant populations (“QMPs vs. Residents”) and across digitization methods (“Manual vs. Solid Applicator”) as indicated by the gold color.

For the manual digitization, mean and standard deviation of occurrence, severity, and detectability ratings were calculated separately for each FMEA row. Each row contains a failure mode and associated cause. This calculation was performed for QMPs and residents separately to compare experience levels with FMEA ratings. The mean OSD parameter values were multiplied together to produce a RPN for each FMEA row (Supplementary Materials). The RPNs for the same failure modes and causes were compared between the two samples: QMPs and residents, using a two‐tailed, unpaired Welch's *t*‐test (*t*‐test “1” in Figure 3). An unpaired test was performed since the respondent groups were not the same and a Welch's test was chosen because the variances (i.e., standard deviations) of the groups were not assumed to be equal. All t‐tests in this study were performed with GraphPad Prism software with *α* = 0.05. FMEA data analysis consisted of multiple *t*‐tests as illustrated in Figure 3.

For the solid applicator method data, mean and standard deviation OSD values were similarly calculated, and then multiplied together to form RPNs for QMPs and residents separately. The RPN distributions of the unique steps were statistically compared across digitization methods for both participant samples using separate two‐tailed, unpaired Student's *t*‐tests (“2,3”). An unpaired method was selected because the unique steps of the solid applicator method do not directly correlate to the unique steps of the manual method. In other words, the test is not analyzing the same method before and after a change—in which case, a paired test would be most appropriate. Finally, two‐tailed, unpaired Student's *t*‐tests were performed across methods for each OSD parameter rated by QMPs to isolate the contributions of each rating to the overall RPN results (“4‐6”).

#### 2.7.3 Time data analysis

Four groups of time data: manual digitization times in clinic, manual digitization times on anonymized patients, and solid applicator digitization times (first and second attempts), were statistically compared using a one‐way ANOVA test. This test was performed with no pairing and on GraphPad Prism software. Tukey's test was subsequently performed with the same software to determine which groups had statistically significant means. First and second solid applicator times were analyzed independently of each other as familiarity and efficiency were expected to improve with repetition. Thus, the second times were expected to be a better representation of the time to digitize after institution's adoption of the technique.

To quantify the effect of environment (i.e., in‐ vs. out‐of‐clinic) on digitization while minimizing participant sample variation, a paired calculation was performed for participants who were timed on manual digitization for both patient samples. To begin, each participant's manual anonymized digitization time was subtracted from their average in‐clinic time. From these differences, mean and standard deviation were calculated across participants.

Finally, separate GraphPad Prism simple linear regressions were performed for manual digitization times for anonymized patients and second solid applicator digitization times. These data sets have the same environment, patient sample, and participant sample. Specifically, the regression *p*‐value and linearity (R^2^) were analyzed between digitization time and years of brachytherapy experience.

## 3 RESULTS

### 3.1 Participant experience

Overall, the brachytherapy experience of participants ranged from 1 to 13 years with an average of 5.6 ± 4.0 years. Seven of eight participants expressed familiarity with solid applicator digitization for treatment planning beyond the complexity of the vaginal cylinder applicator (“complex applicators”). Two of the seven participants had digitized complex solid applicators clinically at previous institutions, while the other five had worked with complex solid applicator digitization in non‐clinical settings. Of the eight participants, two had performed FMEA for clinical procedure assessment prior to this study.

### 3.2 FMEA scores

#### 3.2.1 Manual method

For manual digitization, there were 63 identified failure modes/causes each with an associated RPN. For QMPs, the RPN mean and standard deviation were 20.7 ± 10.3. For these same FMEA failure modes and causes as rated by residents, the mean and standard deviation were 53.0 ± 52.2. Further, the maximum RPN was 46.3 for QMPs and 283.5 for residents. When comparing between QMP and resident respondents, there was a statistically significant difference in RPN means (*p*‐value < 0.001).

#### 3.2.2 Method comparison

**FIGURE 4:**
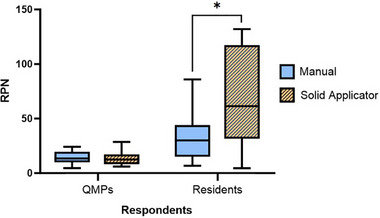
FMEA RPN scores across manual (blue) and solid applicator (orange, striped) digitization for QMPs (left) and residents (right). QMPs found no significant difference between the digitization method RPN means, while residents found the solid applicator method to have a higher mean. Asterisk (*) indicates significance: *p*‐value < 0.05, between the indicated groups.

Between the two methods, 19 unique failure modes and effects were isolated for manual digitization and 20 were isolated for solid applicator digitization. QMPs ratings did not find significance between RPN means of these unique FMEA rows (*p*‐value = 0.70), while residents did (*p*‐value = 0.003) as shown in Figure 4. From resident responses only, the mean RPN for the solid applicator method was twice as large as that of the manual method (68.2 ± 21.7 and 32.6 ± 44.0, respectively).

**FIGURE 5:**
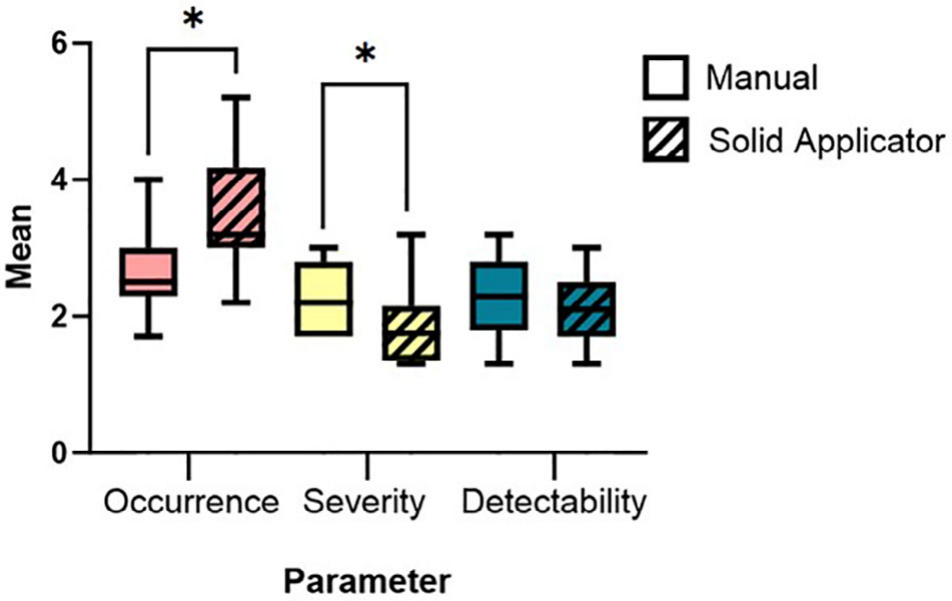
Occurrence (left), severity (middle), and detectability (right) scores across unique method steps as rated by QMPs. Statistical significance was found for two of the three parameters with the solid applicator method failures rated more likely to occur, but with lower severity. Asterisk (*) indicates significance: *p*‐value < 0.05, between the indicated groups.

QMP ratings for OSD parameters of the 39 total unique failure modes and effects are shown across the manual and solid applicator methods in Figure 5. The occurrence and severity of failures were statistically different between the methods (*p*‐value = 0.002 and 0.04, respectively). Detectability of failures was not significantly different (*p*‐value = 0.09). For the solid applicator method, the mean occurrence was higher than that of the manual method while the mean severity and detectability were lower.

### 3.3 Time to digitize

**FIGURE 6:**
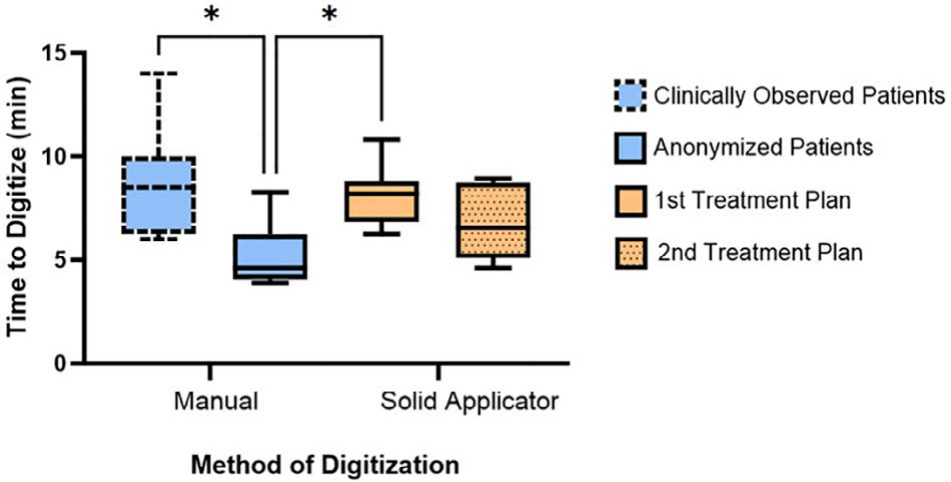
All collected digitization times are separated by the method of digitization (manual vs. solid applicator) and by patient/plan category. Dashed and solid line patterns indicate timing instrument: minute resolution clock and stopwatch, respectively. Digitization using the manual method on anonymized patients took statistically less time than two other patient/plan groups. There was no significance found between manual anonymized patient and solid applicator second treatment plan digitization. Asterisk (*) indicates significance: *p*‐value < 0.05, between the indicated groups.

All R&T digitization plan times are represented in Figure 6. Statistical significance was shown using ANOVA between at least two of the means (*p*‐value = 0.005). Tukey's test found significant differences in time for manual digitization on anonymized patients against both manual digitization on clinically observed patients (adjusted *p*‐value = 0.005) and first solid applicator plans (adjusted *p*‐value = 0.025).

The four participants timed on both manual patient sets showed a mean reduction of 3.92 ± 1.06 min from clinically observed to anonymized patients. All of these participants showed a mean reduction greater than two minutes. Between the first and second solid applicator treatment plans, there was an average decrease of 1.27 ± 1.58 min. Not all participants showed reductions in time, but five of eight that did were able to digitize the second plan over a minute faster than their first plan. On a patient‐basis, each anonymized treatment plan had three time points collected: manual digitization and solid applicator digitization times (first and second attempts). No single treatment plan possessed the two highest or two lowest time points collected overall which could indicate potential outliers in the data sets.

**FIGURE 7:**
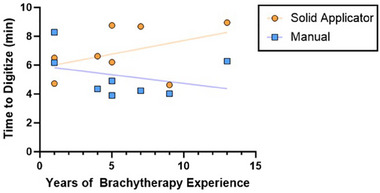
Simple linear regression of time of digitization across both digitization methods and all participants by years of brachytherapy experience. Second treatment plan solid applicator digitization (orange circles) has a R^2^ = 0.1903 and equation of best fit of Y = 0.1890*X + 5.819. Anonymized patient manual digitization (blue squares) has a R^2^ = 0.1018 and equation of best fit of Y = −0.1210*X + 5.945. Residents (1 year of brachytherapy experience) performed solid applicator digitization faster than the manual method, while the opposite was found for QMPs (4–13 years).

Finally, the linear best fit between years of experience and digitization time are shown in Figure 7 for each method and all participants. The regression p‐values were not significant at values of 0.44 and 0.28 for manual and solid applicator digitization, respectively. Further, the R^2^ value was 0.1018 for the manual method and 0.1903 for the solid applicator method.

## 4 DISCUSSION

The aim of this study is to quantify the practicality of solid applicator R&T digitization use at our institution based on risk and time metrics. Previous gynecological brachytherapy studies have found that the FMEA protocol recommended by TG‐100 was a comprehensive evaluation from applicator placement to treatment delivery.^12^, ^13^, ^14^, ^15^ Thus, this study consisted of the scoring of two applicator digitization FMEAs (i.e., manual and solid applicator methods) by QMPs and residents involved in brachytherapy treatment planning at our institution. For the 60+ determined failure modes/causes in the manual digitization (see Supplementary Materials), the difference in RPN means were statistically significant between ratings of QMPs and residents. For this manual method, the resident rated RPN mean, standard deviation, and maximum were higher than those rated by QMPs. This increase is likely due to a difference in brachytherapy experience, and this finding supports the individual evaluation of FMEA responses from QMPs and residents for the method comparison portion of this study.

Notably, even with resident responses included, the maximum RPN found in this study for manual digitization (i.e., 79.2) did not exceed the maximum RPN found in similar research on a R&T workflow FMEA (i.e., 192).^12^ The difference in maximums may be a result of the method of RPN calculation (i.e., in this study OSD values are first averaged across participants) or as a result of the portion of the workflow observed by the studies (i.e., only applicator digitization as opposed to applicator insertion through treatment delivery). Further, the lower maximum of this study could be attributed to different workflow verification checks which would require further inter‐institutional analysis.

There was a minimal difference in the number of unique failure modes/causes found for manual and solid applicator digitization (19 vs. 20). Across the methods, QMP respondents found no significant difference in RPNs. This non‐significance indicates that the adoption of the solid applicator method at our institution for R&T digitization may not change the risk of workflow failures. Although resident respondents planned more quickly using the solid applicator method than with the manual method, these participants also rated the solid applicator digitization with a significantly higher RPN mean. This may be due to less experience with assessing the risk of a new method and may be magnified by the low number of residents surveyed (i.e., higher scores from one participant would enlarge the mean by a greater margin). Based on these limitations in resident responses, QMP FMEA results are likely more reliable to base institutional changes upon.

Subsequent analysis of QMP OSD parameters found that the occurrence of failures was more likely in the solid applicator method, while the severity of failures was lower using the solid applicator models. The heightened occurrence is expected since this is a new protocol for the participants, so the perception of making mistakes may be more likely. The lower severity may be due to the pre‐defined nature of the solid models. The non‐significance of the final parameter: detectability, may be a result of using the same treatment planning system (i.e., BrachyVision) between the methods.

With the adoption of the solid applicator protocol, OSD parameters may change over time. Notably, occurrence scores would likely decrease due to repetition and familiarity. The severity and detectability scores of the solid applicator method may change less through the adoption process, but the means of both these risk parameters were already lower in this study than the associated manual digitization values. Thus, according to QMP results, solid applicator protocol implementation is feasible from a risk standpoint.

In terms of time, brachytherapy is a unique subset within radiation oncology procedures, requiring real‐time planning since the position of applicators may shift over time. Thus, efficient treatment planning can help to reduce discrepancies between the applicator position on initial CT scans and the applicator position at the time of treatment. One step of this brachytherapy treatment planning process is applicator digitization. This study found a statistically lower manual digitization time recorded for anonymized patient cases as opposed to those observed in the clinic. This reduction is further supported by the average reduction of nearly 4 (± 1.06) min that was found across the four participants that digitized both patient populations. This significant difference is unexpected because clear distractions (i.e., participant focus shifted away from digitization) were noted and removed for in‐clinic timing. The remaining difference in time may be due to the nonidentical timing instruments used in data collection, multitasking in the clinic that was not omitted from digitization time, or additional caution on plans that the participant knows will be delivered to a patient.

While there was not a significant difference between first and second solid applicator treatment plan times, the mean did decrease with repetition as expected for the majority of participants (i.e., 5/8). The mean of the first solid applicator times was significantly higher than the mean for the manual method on the same patient population. However, after the completion of the participants’ second solid applicator plans, time was not significantly different between the two digitization methods. While it is difficult to predict the extent to which the solid applicator method times will further decrease, the non‐inferiority of the second plan with the current method is promising for potential clinical adoption. Based on the second plan times, implementation of the solid applicator method would be feasible at our institution without compromising treatment time. Further investigation could be performed after additional solid applicator digitization to test for significance between methods after repetition.

Further, manual digitization times decreased slightly over years of experience and while solid applicator times increased. While these slopes may indicate the tradeoff between experience and malleability, the *p*‐values were not significant indicating a poor correlation. Further, the low R^2^ measures indicate that linearity did not well represent the relationship between experience and planning time. In addition to the slight slope trends observed in Figure 7, the residents were the only respondents who performed solid applicator digitization faster than manual digitization. This speed after two attempts points to the resident's adaptability with the new protocol and supports the feasibility of training new members of the physics team using solid applicator methods.

The time analysis of this study is optimistic toward the implementation of R&T solid applicator digitization based on the average reduction in time between solid applicator plans and the non‐significant time difference between methods. Additionally, non‐inferiority of FMEA failure modes was found by QMPs further supporting the suitability of implementation. Limitations to this study include the small, but diverse participant sample. This makes it difficult to group participants by experience (e.g., small subsamples of QMPs with complex solid applicator experience). It would be recommended that additional medical physicists and institutions are surveyed using the described FMEAs and time analyses in this study. Further practice performing the solid applicator protocol is recommended for the original participants of this study as well as optional adjustments to their FMEA scores over time. Results could be tested for significance to determine if participants gain confidence or a different perspective of the process with repetition.

Expanding toward new vendor treatment planning protocols also warrants investigation into using plan templates for R&T cases where time may be saved with pre‐defined reference points relative to the applicators—a step not covered in this study. Further, this study prompts an investigation into the method of digitization taught to residents. Resident digitization times favored the solid applicator method despite each resident previously completing a clinical rotation using manual digitization. Thus, the solid applicator method may be adopted easier and/or quicker by residents than the manual method. The pre‐defined geometric constraints of solid applicator models may also reduce the time needed for QMP verification of accurate digitization—a step that was not explored in this study. Finally, this work could be built upon an analysis of planned and delivered dose distributions using solid applicator and manual R&T digitization to determine potential differences in the accuracy of brachytherapy treatment delivery between the digitization methods.

## 5 CONCLUSION

FMEA and digitization time data were collected across six QMPs and two residents involved in brachytherapy treatments at our institution. This study used FMEA to quantify the occurrence, severity, and detectability of risks in the manual digitization of R&T brachytherapy applicators. Produced RPN values were compared to a second FMEA which evaluated digitization risk using solid applicator models. Resident respondents found a higher mean RPN for the solid applicator method, while QMPs found no significant difference in RPN means between the digitization methods. QMPs rated the occurrence of failure higher and the severity of failure lower for the solid applicator method as compared to manual digitization. Across all participants, there was no significant difference in digitization time between the two methods. The combined risk and time findings of this study demonstrate the non‐inferiority of the developed solid applicator protocol and the viability of clinical implementation.

## CONFLICT OF INTEREST STATEMENT

The authors declare no conflicts of interest.

## Supporting information

Supporting Information

Supporting Information

Supporting Information

Supporting Information

Supporting Information

Supporting Information

Supporting Information
