# Supplementary figures and images for: Comparison of failure modes and effects analyses and time for brachytherapy ring and tandem applicator digitization between manual and solid applicator source placement methods
Source: J Appl Clin Med Phys. 2024 Apr 25;25(5):e14336. doi: 10.1002/acm2.14336 (PMC11087182; doi:10.1002/acm2.14336)

Process Map - Solid Applicator Digitization

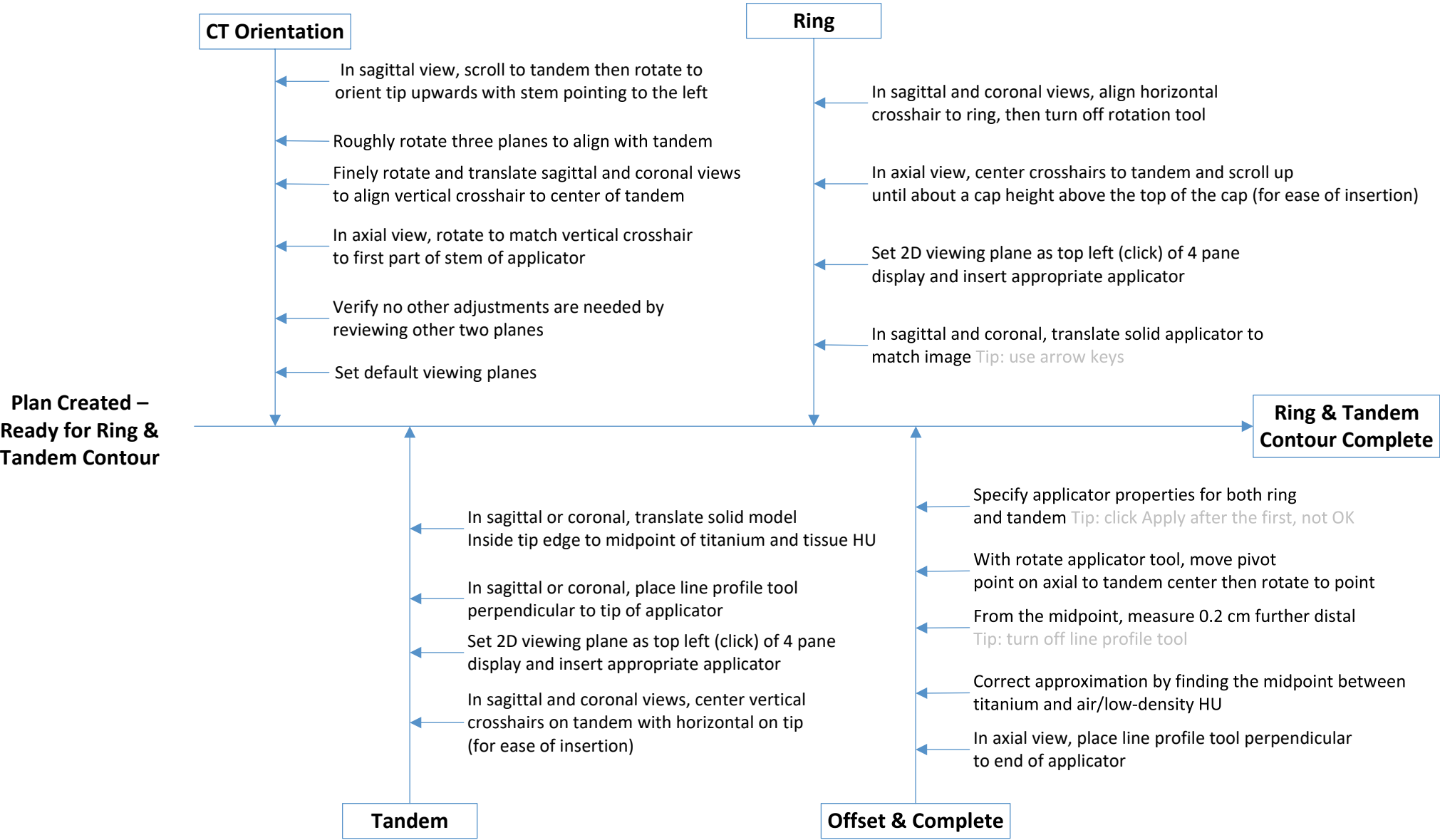

Supplement: Supplementary file 1 — Supporting Information [file ACM2-25-e14336-s004.pdf]

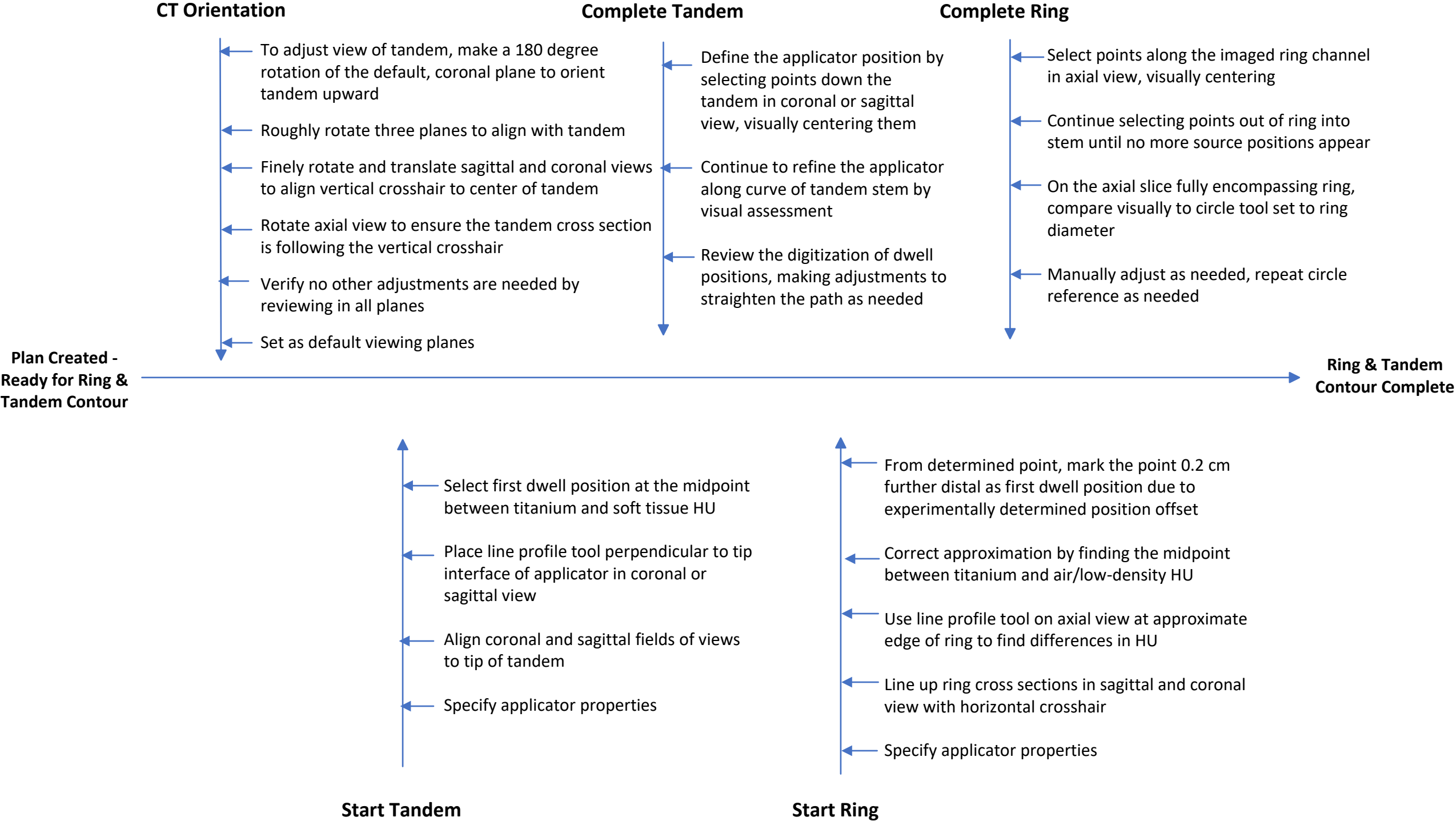

Supplement: Supplementary file 2 — Supporting Information [file ACM2-25-e14336-s003.pdf]
