# Supplementary material for: Comparison of failure modes and effects analyses and time for brachytherapy ring and tandem applicator digitization between manual and solid applicator source placement methods
Source: J Appl Clin Med Phys. 2024 Apr 25;25(5):e14336. doi: 10.1002/acm2.14336 (PMC11087182; doi:10.1002/acm2.14336)
Supplement: Supplementary file 3 — Supporting Information [file ACM2-25-e14336-s001.pdf]

FMEA REFERENCE SHEET (Adapted from Beth Isael Lahey Health Radiation Oncology & AAPM TG-100)

Risk rating scales for process FMEA (AAPM TG 100 Table II)
AAPM TG 100, Table II

Score	Occurrence (*O*)		Severity (*S*)		Detectability Risk (*D*)	
	Qualitative	Frequency in %	Qualitative	Categorization	Qualitative	Probability not detected in %
**1**	*Failure unlikely*	0.01	*No effect*	*No effect*	*Obvious*	0.01
**2**		0.02	*Inconvenience*	*Inconvenience*		0.2
**3**	*Relatively Few Failures*	0.05			*Easy to detect*	0.5
**4**		0.1	*Minor dosimetric error*	*Suboptimal plan or treatment*		1.0
**5**		< 0.2	*Limited toxicity or target underdose*	*Wrong dose, dose distribution, location, or volume*		2.0
**6**	*Occasional Failures*	< 0.5			*Detectable with careful review*	5.0
**7**		< 1	*Possible serious toxicity or target underdose*			10
**8**	*Repeated Failures*	< 2			*Only detectable with thorough investigation*	15
**9**		< 5	*Possible **very** serious toxicity or target underdose*	***Very** wrong dose, dose distribution, location, or volume*		20
**10**	*Failures Inevitable*	> 5	*Catastrophic*		*Impossible to detect*	> 20

**End Effect**	**Description**
Planning Delay	Time delay induced to later physicist step in reconstruction
Treatment Delay	Time delay induced to later RadOnc team step in patient procedure
Reconstruction Deviation	Differences in physical and digital applicator geometry due to poor alignment (inaccurate) or inter-user variability (inconsistent)
Treatment Deviation	Differences in expected and delivered treatment due to non-reconstruction influences

Suggested severity score ranges (AAPM TG-100, Table I)

**Severity (S)**		
**Error**	**Score Range**	
Suboptimal plan delivery	**4**	
Non-radiation physical injury	**5**	**10**
Inconvenience (patient)	**2**	**3**
Inconvenience (staff/cost)	**1**	**2**
