# Supplementary material for: Comparison of failure modes and effects analyses and time for brachytherapy ring and tandem applicator digitization between manual and solid applicator source placement methods
Source: J Appl Clin Med Phys. 2024 Apr 25;25(5):e14336. doi: 10.1002/acm2.14336 (PMC11087182; doi:10.1002/acm2.14336)
Supplement: Supplementary file 4 — Supporting Information [file ACM2-25-e14336-s006.pdf]

Ring & Tandem Manual Digitization (1) FMEA

Radiation Oncology (spreadsheet tool adapted from Amanda Swanson (OSU) and template provided by BI-Lahey Health | Radiation Oncology & AAPM WG 100)

** This is an example FMEA with average OSD values from all participants rather than the open OSD boxes that were filled in during the study.

Please enter O/S/D scores for each failure mode on a scale from 1-10.

Thank you for participating!

Section #	Process Steps	Step #	# of Potential Failure Modes	Potential Failure Mode	# of Potential Causes	Potential Causes of Failure	End Effect	O (Mean)	O (St Dev)	S (Mean)	S (St Dev)	D (Mean)	D (St Dev)	RPN	Comments
1	CT Orientation														
	To adjust view of tandem, make a 180 degree rotation of the default, coronal plane to orient tandem upward	1.0	2	Poor image alignment to initiate planning	1	Plane rotated to incorrect location (too litte/far)	Planning Delay	2.6	1.2	2.0	1.1	2.3	1.4	11.8	
				Coronal plane not rotated	2	Incorrect, intended plane rotated	Planning Delay	2.3	1.5	2.0	1.1	2.0	2.1	9.0	
						No plane rotated (step forgotten)	Planning Delay	1.6	0.7	2.0	1.1	1.6	0.7	5.3	
	Roughly rotate three planes to align with tandem	1.1	1	Poor image alignment to initiate planning	1	Focus on only one or two planes	Planning Delay	2.9	1.2	2.6	0.9	2.1	1.0	16.0	
	Finely rotate and translate sagittal and coronal views to align vertical crosshair to center of tandem	1.2	2	Rotation of tandem in one or both plane	1	Human error (lack of check in all planes after a rotation/translation)	Digitization Deviation	2.8	1.0	2.8	1.2	3.4	2.0	25.5	
				Translation of tandem from image in one or both planes	1	Human error (lack of check in all planes after a rotation/translation)	Digitization Deviation	2.8	1.0	2.9	1.7	2.9	1.7	22.7	
	Rotate axial view to ensure the tandem cross section is following the vertical crosshair	1.3	1	Rotation in axial plane	1	Too little or too much emphasis on the stem as opposed to the tandem itself	Digitization Deviation	3.0	1.4	2.0	0.8	3.0	2.3	18.0	
	Verify no other adjustments are needed by reviewing in all planes	1.4	1	Rotation or translation in other planes	1	Assumption of accuracy, skip quality check step	Digitization Deviation	2.9	1.2	3.0	2.3	3.5	2.4	30.2	
	Set as default viewing planes	1.5	2	Default image planes not saved for reference	1	Step forgotten	Planning Delay	5.4	2.6	2.1	1.0	1.3	0.5	14.3	
				Default image planes incorrectly set	1	Selection error to System Default viewing planes	Planning Delay	1.6	0.7	2.1	1.0	1.1	0.4	3.9	
2	Start Tandem														
	Specify applicator properties	2.0	7	Incorrect Channel	2	Incorrect value by accident	Treatment Deviation	2.4	0.9	8.6	1.3	3.3	1.8	66.6	
						Institution/System Conventions Not Known	Treatment Deviation	2.6	1.9	8.6	1.3	3.5	2.3	79.2	
				Incorrect Channel Length	3	Do not change	Treatment Delay	3.0	0.9	4.6	3.0	2.9	1.7	39.9	
						Incorrect value by accident	Treatment Delay	3.0	1.2	4.6	3.0	3.0	2.0	41.6	
						Institution/System Conventions Not Known	Treatment Delay	3.5	1.5	4.6	3.0	3.3	2.3	52.6	
				Do not change First Source Position	1	Do not change	Treatment Deviation	2.8	1.0	2.9	1.6	3.0	2.4	23.7	
				Change First Source Position Incorrectly	2	Incorrect value by accident	Treatment Deviation	2.3	0.9	5.5	2.8	2.8	1.0	34.0	
						Institution/System Conventions Not Known	Treatment Deviation	2.6	1.1	5.5	2.8	2.5	1.2	36.1	
				Do not change Last Source Position	1	Do not change	Treatment Deviation	1.4	0.7	4.3	3.5	1.3	0.7	7.3	
				Change Last Source Position Incorrectly	2	Incorrect value by accident	Treatment Deviation	2.3	1.2	4.1	2.4	2.3	1.0	20.9	
						Institution/System Conventions Not Known	Treatment Deviation	2.4	1.3	4.1	2.4	2.1	1.0	20.8	
				Non-Adjusted Property Incorrect (Afterloader, Equal Source Spacing, Dead Space)	3	Accidental Selection Error	Treatment Deviation	1.4	0.7	4.3	2.3	3.0	2.2	17.5	
						Not checked (incorrect preset missed)	Treatment Deviation	2.0	1.4	4.3	2.3	3.1	2.5	26.6	
						Institution/System Conventions Not Known	Treatment Deviation	2.0	1.1	4.3	2.3	2.8	1.5	23.4	
	Align coronal and sagittal field of views to tip of tandem	2.1	1	Vertical crosshair not centered on both planes	1	Not checked / considered	Digitization Deviation	2.3	1.2	3.0	1.1	3.6	2.3	24.5	
						Only one plane adjusted	Digitization Deviation	2.6	1.1	3.0	1.1	3.1	1.7	24.6	
	Place line profile tool perpendicular to tip interface of applicator in coronal or sagittal view	2.2	2	Line not perpendicular to tip and centered on tandem	1	Not checked / considered	Digitization Deviation	2.9	0.8	2.8	1.6	3.3	2.1	25.7	
				Tool not used	1	Step skipped	Digitization Deviation	1.9	1.0	3.9	1.5	3.8	2.5	27.2	
	Select first dwell position at the midpoint between titanium and soft tissue HU	2.3	1	Too far toward soft tissue or titanium HU	2	No objective, persisting rule	Digitization Deviation	4.1	2.9	2.5	1.4	3.5	1.6	36.1	
						Influenced by visual appearance of tip	Digitization Deviation	3.8	1.4	2.5	1.4	4.8	2.8	44.5	

3	**Complete Tandem**														
	Define the applicator position by selecting points down the tandem in coronal or sagittal view, visually centering them	3.0	1	Deviations from tandem geometry	1	Human error	Digitization Deviation	2.9	0.8	3.4	1.4	2.5	1.2	24.3	
	Continue to refine the applicator along curve of tandem stem by visual assessment	3.1	1	Deviations from tandem geometry	2	Too few points used to represent curvature well	Digitization Deviation	3.1	1.5	2.0	1.1	2.3	1.0	14.1	
						Direction of points does not follow applicator	Digitization Deviation	2.4	1.1	2.0	1.1	1.8	0.9	8.3	
	Review the digitization of dwell positions, making adjustments to straighten the path as needed	3.2	2	Inconsistent determination by physicist	1	No straight line reference check to refer to	Digitization Deviation	2.6	1.4	3.0	1.2	4.1	2.6	32.5	
				No review of positions	1	Step skipped	Digitization Deviation	2.5	1.2	3.4	1.3	3.5	2.9	29.5	
4	**Start Ring**														
	Specify applicator properties	4.0	5	Incorrect Channel	2	Incorrect value by accident	Treatment Deviation	2.4	0.9	8.6	1.3	3.3	1.8	66.6	
						Institution/System Conventions Not Known	Treatment Deviation	2.6	1.9	8.6	1.3	3.5	2.3	79.2	
				Incorrect Channel Length	3	Do not change	Treatment Delay	3.0	0.9	4.6	3.0	2.9	1.7	39.9	
						Incorrect value by accident	Treatment Delay	3.0	1.2	4.6	3.0	3.0	2.0	41.6	
						Institution/System Conventions Not Known	Treatment Delay	3.5	1.5	4.6	3.0	3.3	2.3	52.6	
				Do not change First Source Position	1	Do not change	Treatment Deviation	2.8	1.0	2.9	1.6	3.0	2.4	23.7	
				Change First Source Position Incorrectly	2	Incorrect value by accident	Treatment Deviation	2.3	0.9	5.5	2.8	2.8	1.0	34.0	
						Institution/System Conventions Not Known	Treatment Deviation	2.6	1.1	5.5	2.8	2.5	1.2	36.1	
				Do not change Last Source Position	1	Do not change	Treatment Deviation	1.4	0.7	4.3	3.5	1.3	0.7	7.3	
				Change Last Source Position Incorrectly	2	Incorrect value by accident	Treatment Deviation	2.3	1.2	4.3	2.4	2.3	1.0	21.5	
						Institution/System Conventions Not Known	Treatment Deviation	2.4	1.3	4.3	2.4	2.1	1.0	21.4	
				Non-Adjusted Property Incorrect (Afterloader, Equal Source Spacing, Dead Space)	3	Accidental Selection Error	Treatment Deviation	1.4	0.7	4.3	2.3	3.0	2.2	17.5	
						Not checked (incorrect preset missed)	Treatment Deviation	2.0	1.4	4.3	2.3	3.1	2.5	26.6	
						Institution/System Conventions Not Known	Treatment Deviation	2.0	1.1	4.3	2.3	2.8	1.5	23.4	
	Line up ring cross sections in sagittal and coronal view with horizontal crosshair	4.1	1	Horizontal crosshair not centered on both planes	1	Not checked	Digitization Deviation	2.8	1.4	3.6	1.3	2.8	1.4	27.4	
	Use line profile tool on axial view at approximate edge of ring to find differences in HU	4.2	1	Line tool not drawn parallel and centered on ring	1	Not checked / considered	Digitization Deviation	2.9	1.6	2.9	0.8	3.5	2.1	28.9	
	Correct approximation by finding the midpoint between titanium and air/low-density HU	4.3	1	Inconsistent determination by physicist	1	No objective, persisting rule	Digitization Deviation	2.9	1.6	2.8	0.9	3.5	2.1	27.7	
	From determined point, mark the point 0.2 cm further distal as first dwell position due to experimentally determined offset of positons	4.4	2	Distance incorrectly measured or not measured at all	1	Not checked / considered	Digitization Deviation	3.0	1.4	3.9	1.2	3.5	1.5	40.7	
				Extrapolation angle not consistent with ring	1	Not checked / considered	Digitization Deviation	3.3	1.8	2.4	1.1	3.1	1.7	24.1	
5	**Complete Ring**														
	Select points along the imaged ring channel in axial view, visually centering	5.0	1	Deviations from circle	2	Visual alignment error	Digitization Deviation	2.9	1.0	3.0	1.1	3.4	1.8	29.1	
						Insufficient points used	Digitization Deviation	2.1	0.8	3.1	1.1	2.5	1.4	16.6	
	Continue selecting points out of ring into stem until no more source positions appear	5.1	1	Curve not accurately reconstructed	1	No quality check for moving between planes through curve	Digitization Deviation	3.0	1.1	2.1	1.4	2.3	1.0	14.3	
	On the axial slice fully encompassing ring, compare visually to circle tool set to ring diameter	5.2	3	Wrong slice used	1	Not checked / considered	Digitization Deviation	3.5	2.8	2.3	0.9	3.1	1.9	24.6	
				Wrong size of circle tool used	2	Mistype	Digitization Deviation	3.5	2.9	1.9	0.6	2.0	1.2	13.1	
						Do not change from default	Digitization Deviation	3.3	3.1	1.9	0.6	1.9	1.1	11.4	
				Incorrect centering of circle template	1	Influenced by manual placement of points	Digitization Deviation	3.5	2.9	1.9	0.6	2.1	1.2	13.9	
	Manually adjust as needed, repeat circle reference as needed	5.3	2	Adjusted inaccurately	2	Over/under compensation	Digitization Deviation	2.0	0.9	2.8	1.2	3.1	2.4	17.2	
				Not repeated sufficiently	1	False assumption of accuracy	Digitization Deviation	2.5	1.4	2.6	1.3	3.1	2.4	20.5	
