# Supplementary material for: Comparison of failure modes and effects analyses and time for brachytherapy ring and tandem applicator digitization between manual and solid applicator source placement methods
Source: J Appl Clin Med Phys. 2024 Apr 25;25(5):e14336. doi: 10.1002/acm2.14336 (PMC11087182; doi:10.1002/acm2.14336)
Supplement: Supplementary file 5 — Supporting Information [file ACM2-25-e14336-s007.pdf]

Ring & Tandem Solid Applicator Digitization (2) FMEA

Radiation Oncology (spreadsheet tool adapted from Amanda Swanson (OSU) and template provided by BI-Lahey Health | Radiation Oncology & AAPM WG 100)

** This is an example FMEA with average OSD values from QMPs rather than the open OSD boxes that participants filled in.

Please enter O/S/D scores for each failure mode on a scale from 1-10.

Thank you for participating!

Section #	Process Steps	Step #	# of Potential Failure Modes	Potential Failure Mode	# of Potential Causes	Potential Causes of Failure	End Effect	O (Mean)	O (St Dev)	S (Mean)	S (St Dev)	D (Mean)	D (St Dev)	RPN	Comments	Associated Step in FMEA 1 (Numbers Represent Excel Row Numbers)
1	**CT Orientation**															
	In sagittal view, scroll to tandem then rotate to orient tip upwards with stem pointing to the left	1.0	2	Poor image alignment to initiate planning	1	Plane rotated to incorrect location (too little/far)	Planning Delay	3.2	1.2	1.5	0.5	2.5	1.5	11.9		
				Sagittal plane not rotated	2	Incorrect plane rotated	Planning Delay	3.2	1.2	1.3	0.8	2.0	1.7	8.4		
						No plane rotated (step forgotten)	Planning Delay	2.5	1.4	1.3	0.8	2.0	1.7	6.7		
	Roughly rotate three planes to align with tandem	1.1	1	Poor image alignment to initiate planning	1	Focus on only one or two planes	Planning Delay									13
	Finely rotate and translate sagittal and coronal views to align vertical crosshair to center of tandem	1.2	2	Rotation of tandem in one or both plane	1	Human error (lack of check in all planes after a rotation/translation)	Digitization Deviation									14
				Translation of tandem from image in one or both planes	1	Human error (lack of check in all planes after a rotation/translation)	Digitization Deviation									15
	In axial view, rotate to match vertical crosshair to first part of stem of applicator	1.3	1	Rotation in axial plane	1	Too little or too much emphasis on the stem as opposed to the ring itself	Digitization Deviation									16
	Verify no other adjustments are needed by reviewing other two planes	1.4	1	Rotation or translation in other planes	1	Assumption of accuracy, skip quality check step	Digitization Deviation									17
	Set default viewing planes	1.5	2	Default image planes not saved for reference	1	Step forgotten	Planning Delay									18
				Default image planes incorrectly set	1	Selection error to System Default viewing planes	Planning Delay									19
2	**Tandem**															
	In sagittal and coronal views, center vertical crosshairs on tandem horizontal on tip (for ease of insertion)	2.0	2	Vertical crosshair not centered on both planes	1	Not checked / considered	Planning Delay	2.8	0.8	1.5	0.8	2.3	1.6	9.9		
				Horizontal crosshair not aligned	1	Not checked / considered	Planning Delay	3.0	1.5	1.3	0.8	1.5	0.8	6.0		
	In sagittal or coronal, place line profile perpendicular to tip of applicator	2.1	2	Line not perpendicular to tip and centered on tandem	1	Not checked / considered	Digitization Deviation									38
				Tool not used	1	Step skipped	Digitization Deviation									39
	In sagittal or coronal, determine midpoint of titanium and tissue HU	2.2	1	Too far toward soft tissue or titanium HU	2	No objective, persisting rule	Digitization Deviation									40
						Influenced by visual appearance of tip	Digitization Deviation									41
	Set 2D viewing plane as top left (click) of 4 pane display and insert appropriate applicator	2.3	2	Do not click top left pane	1	Not checked / considered	Planning Delay	5.2	2.6	1.5	0.8	1.3	0.8	10.3		
				Incorrect applicator chosen	2	Accidental Selection Error	Planning Delay	4.7	2.8	2.0	1.3	1.8	0.8	17.1		
						Misunderstanding of Applicator Type	Planning Delay	3.8	2.1	2.0	1.3	2.5	1.4	19.2		
	Verify placement of solid applicator inside tip edge to midpoint	2.4	1	Inside tip not aligned to line profile mark	1	Outside tip edge placed at line profile mark	Digitization Deviation	2.5	1.0	2.2	1.2	2.7	1.9	14.4		
3	**Ring**															
	In sagittal and coronal views, align horizontal crosshairs to ring, then turn off rotation tool	3.0	2	Horizontal crosshair not centered on both planes	1	Not checked / considered	Digitization Deviation									65
				Rotation tool not turned off	1	Not checked / considered	Planning Delay	3.7	1.4	1.3	0.5	1.7	0.8	8.1		
	In axial view, center crosshairs to ring using circle tool and set axial plane about a cap height above the top of the cap (for ease of insertion)	3.1	2	Crosshairs not centered on axial plane to ring	1	Not checked / considered	Planning Delay	3.2	0.8	1.5	0.8	1.7	0.8	7.9		
				Axial plane height not adjusted correctly	1	Not checked / considered	Planning Delay	3.5	1.0	1.3	0.5	1.5	0.8	7.0		
	Set 2D viewing plane as top left (click) of 4 pane display and insert appropriate applicator	3.2	2	Do not click top left pane	1	Not checked / considered	Planning Delay	5.2	2.6	1.5	0.8	1.3	0.8	10.3		
				Incorrect applicator chosen	2	Misunderstanding of Applicator Type	Planning Delay	4.3	2.7	2.0	1.3	1.8	0.8	15.9		
						Accidental Selection Error	Planning Delay	4.5	2.7	2.0	1.3	2.5	1.4	22.5		

	In sagittal and coronal, translate solid applicator to match image	3.3	1	Not aligned in both planes	1	Not checked / considered	Digitization Deviation	2.2	1.0	3.2	1.6	2.2	1.2	14.9		
4	**Offset & Complete**															
	In axial view, place line profile tool perpendicular to end of applicator	4.0	1	Line not perpendicular to tip and centered on tandem	1	Not checked / considered	Digitization Deviation									66
	Correct approximation by finding the midpoint between titanium and air/low-density HU	4.1	1	Inconsistent determination by physicist	1	No objective, persisting rule	Digitization Deviation									67
	From the midpoint, measure 0.2 cm further distal	4.2	2	Distance incorrectly measured	1	Not checked / considered	Digitization Deviation									68
				Extrapolation angle not consistent with ring	1	Not checked / considered	Digitization Deviation									69
	With circle and rotate applicator tools on, move pivot point on axial to ring center	4.3	3	Circle tool not used	1	Not checked / considered	Digitization Deviation	3.2	1.9	2.2	1.2	2.5	1.9	17.2		
				Circle tool wrong size	1	Not checked / considered	Digitization Deviation	3.0	2.3	2.0	1.3	2.2	0.8	13.0		
				Pivot not moved	1	Not checked / considered	Planning Delay	3.7	2.2	2.3	1.2	2.3	0.8	20.0		
	Rotate applicator to point	4.4	1	Rotated to incorrect position	1	Not checked / considered	Digitization Deviation	3.0	1.3	3.2	1.2	3.0	1.7	28.5		
	Specify applicator properties for ring	4.5	7	Incorrect Channel	2	Incorrect value by accident	Treatment Deviation									22
						Institution/System Conventions Not Known	Treatment Deviation									23
				Incorrect Channel Length	3	Do not change	Treatment Delay									24
						Incorrect value by accident	Treatment Delay									25
						Institution/System Conventions Not Known	Treatment Delay									26
				Do not change First Source Position	1	Do not change	Treatment Deviation									27
				Change First Source Position Incorrectly	2	Incorrect value by accident	Treatment Deviation									28
						Institution/System Conventions Not Known	Treatment Deviation									29
				Do not change Last Source Position	1	Do not change	Treatment Deviation									30
				Change Last Source Position Incorrectly	2	Incorrect value by accident	Treatment Deviation									31
						Institution/System Conventions Not Known	Treatment Deviation									32
				Non-Adjusted Property Incorrect (Afterloader, Equal Source Spacing, Dead Space)	3	Accidental Selection Error	Treatment Deviation									33
						Not checked (incorrect preset missed)	Treatment Deviation									34
						Institution/System Conventions Not Known	Treatment Deviation									35
	Specify applicator properties for tandem	4.6	7	Incorrect Channel	2	Incorrect value by accident	Treatment Deviation									51
						Institution/System Conventions Not Known	Treatment Deviation									52
				Incorrect Channel Length	3	Do not change	Treatment Delay									53
						Incorrect value by accident	Treatment Delay									54
						Institution/System Conventions Not Known	Treatment Delay									55
				Do not change First Source Position	1	Do not change	Treatment Deviation									56
				Change First Source Position Incorrectly	2	Incorrect value by accident	Treatment Deviation									57
						Institution/System Conventions Not Known	Treatment Deviation									58
				Do not change Last Source Position	1	Do not change	Treatment Deviation									59
				Change Last Source Position Incorrectly	2	Incorrect value by accident	Treatment Deviation									60
						Institution/System Conventions Not Known	Treatment Deviation									61
				Non-Adjusted Property Incorrect (Afterloader, Equal Source Spacing, Dead Space)	3	Accidental Selection Error	Treatment Deviation									62
						Not checked (incorrect preset missed)	Treatment Deviation									63
						Institution/System Conventions Not Known	Treatment Deviation									64
