# Supplementary material for: Comparison of failure modes and effects analyses and time for brachytherapy ring and tandem applicator digitization between manual and solid applicator source placement methods
Source: J Appl Clin Med Phys. 2024 Apr 25;25(5):e14336. doi: 10.1002/acm2.14336 (PMC11087182; doi:10.1002/acm2.14336)
Supplement: Supplementary file 6 — Supporting Information [file ACM2-25-e14336-s002.pdf]

All Digitization Time Data

Organized by participant, method, and patient/plan type

	Manual Digitization			Solid Applicator Digitization	
	Clinically Observed	Anonymized		Anonymized	
Participant Identification Number	Time (min)	Treatment Plan	Time (min)	Treatment Plan	Time (min)
1	10	1	4.90	1	8.52
	9			2	8.75
2		1	3.90	1	10.80
				2	6.20
3	7	1	4.02	1	6.55
	6			2	4.63
4	14	1	6.17	1	6.25
	8			2	4.73
5		1	8.28	1	7.75
				2	6.50
6		1	4.35	1	7.88
				2	6.62
7		1	6.27	1	8.85
				2	8.95
8	10	1	4.23	1	8.63
	6			2	8.68
Mean ± Standard Deviation	8.75 ± 2.66		5.27 ± 1.53		7.52 ± 1.68
