# Supplementary material for: Comparison of failure modes and effects analyses and time for brachytherapy ring and tandem applicator digitization between manual and solid applicator source placement methods
Source: J Appl Clin Med Phys. 2024 Apr 25;25(5):e14336. doi: 10.1002/acm2.14336 (PMC11087182; doi:10.1002/acm2.14336)

Solid Applicator Protocol

for Ring & Tandem Reconstruction

Goal 1: Alignment to Tandem

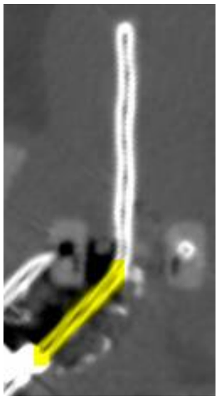

1. In sagittal view, scroll to slice including tandem then **Rotate Display** to point tip of the tandem so that it is pointing upwards and **Pan** (translate) to align tandem to vertical crosshair
2. In coronal view, **Rotate Display** and **Pan** to align tandem to vertical crosshair
3. In axial view, **Rotate Display** and **Pan** to match first part of stem to tandem (See image to right in sagittal. Adjust in axial)
4. Re-check other two planes for any adjustments to ensure alignment
5. Set **Default Viewing Planes**

Goal 2: Place Tandem Solid App.

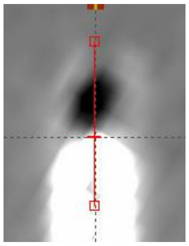

6. In coronal and sagittal planes, center vertical crosshairs on tandem with horizontal crosshair on tip of the tandem
7. In sagittal or coronal plane, place **Line Profile** tool on tip
8. In sagittal or coronal plane, adjust horizontal crosshair to be at the midpoint of titanium and tissue HU (see image to right)
9. Set 2D viewing plane to axial (top left) view and **Insert** appropriate **New Solid Applicator** (see image to right)
   a. Note: Set viewing plane by clicking
   b. Note: New Solid Applicator is ¾ down under Insert tab

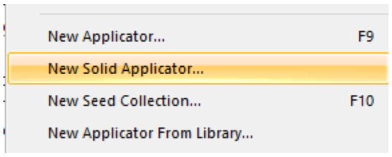

10. Verify placement of solid applicator with inside tip edge to midpoint (see image to right)
    a. Tip: Arrow keys can be used to translate
    b. Note: May need to **Move Applicators** or **Rotate Applicators** slightly if viewing planes were not set up correctly

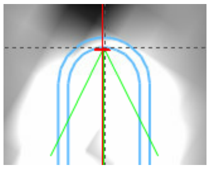

Goal 3: Place Ring Solid App.

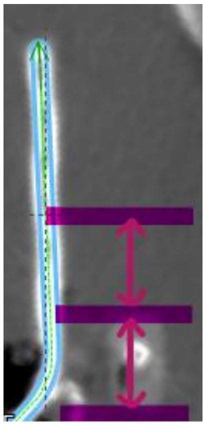

11. In sagittal and coronal planes, align horizontal crosshair to ring with **Rotate Display** tool <u>then turn off rotation tool</u>
12. In axial view, center crosshairs to ring and set horizontal crosshair about one (1) cap height above the top of the cap (see images to right. There is no need to measure)

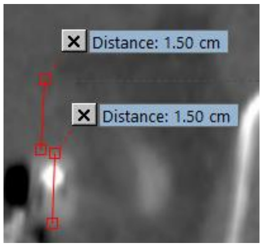

13. Set 2D viewing plane to top left view and insert appropriate **New Solid Applicator**
14. In sagittal and coronal, translate using **Move Applicator** to match image
    a. Tip: Use arrow keys to translate
    b. Note: May need to **Rotate Applicator** slightly if viewing planes were not set up correctly

Goal 4: Offset & Complete

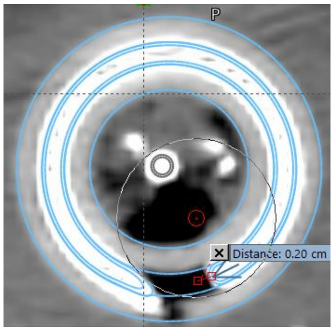

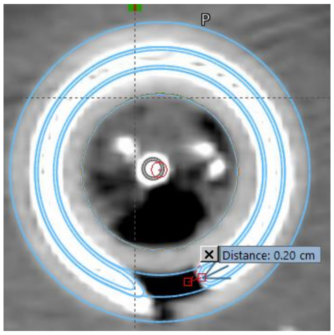

15. In axial view, place **Line Profile** tool on end of ring
16. Determine the midpoint between titanium and air/low-density HU
17. From determined point, **Measure Distance** 0.2 cm into the air/low-density HU space
    a. Tip: Close **Line Profile** tool
18. Turn on **Circle Cursor** tool set to internal cap diameter 1.1 cm and **Rotate Applicator** tool
19. Drag pivot point on axial view to ring center (see images to right for mid-step and completed step expected appearances)
20. In axial view, **Rotate Applicator** to point
21. Right click on the "tandem/ring, channel #" labels in the sidebar to insert applicator **Properties** for both ring and tandem (see image below)
    a. Tip: Click **Apply** after the first are set, not **OK**

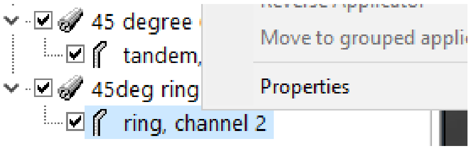

Supplement: Supplementary file 7 — Supporting Information [file ACM2-25-e14336-s005.pdf]
